# Comparison of pulmonary function during interscalene block vs. supraclavicular block: a single-center, double-blind, randomized trial

**DOI:** 10.1186/s12871-022-01967-0

**Published:** 2023-01-10

**Authors:** Jiajia Wang, Xinwei Hou, Xiao Zhang, Xueting Wang, Weiwei Qin, Qiujie Li, Fuguo Ma, Lixin Sun

**Affiliations:** 1 Department of Anesthesiology, Qingdao Municipal Hospital, School of Medicine, Qingdao University, Qingdao, Shandong China; 2 Department of Anesthesiology, Yingkou Central Hospital, Yingkou, Liaoning China

**Keywords:** Brachial plexus block, Pulmonary function, Diaphragm mobility, Diaphragmatic paralysis, Function test, respiratory

## Abstract

**Backround:**

The supraclavicular plexus block (SCB) and interscalene plexus block (ISB) have the potential to pulmonary function, the duration of the potential remains uncertain. So, we compared the effect of SCB and ISB on pulmonary function, especially the duration time.

**Methods:**

Ninety-six patients were finally allocated to group I and group S. The ISB and the SCB procedures were performed with ultrasound guidance before anesthesia induction. An investigator recorded the diaphragm mobility and respiratory function test indicators before the block (T_0_) and at 30 min (T_30 min_), 4 h (T_4_), 8 h (T_8_), and 12 h (T_12_) after the block. The diaphragmatic paralysis rate was calculated for above timepoint. The VAS, the recovery time for the sensory and motor block, and adverse reactions within 24 h of administering the block were also recorded.

**Results:**

The recovery times of diaphragm mobility in group I were longer than those in group S. Compared with group I, group S had a significantly lower diaphragmatic paralysis rate during eupnea breathing at T_30 min_ and T_8_ after the block. Similarly, group S had a significantly lower diaphragmatic paralysis rate at deep breathing at T_30 min_, T_8,_ and T_12_ after the block. The recovery times of FEV_1_ and FVC in group I were longer than those in group S. The other results were not statistically significant.

**Conclusions:**

Ultrasound-guided ISB resulted in a longer periods with a suppressive effect on pulmonary function than SCB.

**Trials registration:**

17/12/2019, ChiCTR1900028286.

## Background

Brachial plexus block has been shown to resolve distal radius fracture-induced strong breakthrough pain [1]. The supraclavicular plexus block (SCB) and interscalene plexus block (ISB) are one of the most commonly used brachial plexus blocking methods. The SCB and ISB are popular with anesthesiologists due to its advantages of low circulation interference, a relatively simple operation, low cost, and postoperative analgesia [2, 3]. In recent years, with the development of ultrasound visualization technology, the success rate of SCB and ISB have increased significantly, and the complications (brachial plexus block, including vascular injury, pneumothorax, Horner syndrome, phrenic nerve palsy and, so on) have been significantly reduced. Even though ultrasound can clearly distinguish the anatomical structure of the brachial plexus and the surrounding tissues, accidental blockage of the phrenic nerve can cause diaphragmatic paralysis [4]. Studies have shown that no matter SCB or ISB is used, the incidence of diaphragmatic muscle block has hit an all-time high [5, 6]. Closer attention is called for since diaphragmatic paralysis caused by phrenic nerve blockage is hard to notice due to the compensatory effect of the contralateral diaphragm [7, 8]. Moreover, long-acting effects of diaphragmatic paralysis persist even when sensory and motor have made a good functional recovery. Pulmonary function changes have only been measured during the short period of clinical trials after induction by the two classical brachial plexus blocks. The time duration of diaphragmatic paralysis caused by brachial plexus block has not been studied.

The diaphragm is the most important respiratory muscle of the human body and it can provide 75% of the resting lung ventilation. Diaphragm paralysis caused by unilateral phrenic nerve block reduces the vital capacity by 20-30% [9, 10]. However, due to the compensation effect, most patients are asymptomatic. Obesity and respiratory disease patients may suffer from respiratory distress due to their decreased respiratory reserve and intolerance of transient diaphragm paralysis [11]. This may even cause respiratory disease due to diaphragm paralysis, which increases the rate of lung infection and affects the quality of life of the patients. Therefore, knowledge of the duration of phrenic nerve block is important, providing a reference for the safety of brachial plexus block selection in clinical application. In the present study, we aimed to compare the effect of SCB and ISB on pulmonary function, especially the duration time.

## Methods

### Study design and randomization

Patients, ASA I or II, aged between 18 and 65 years, undergoing radius fracture surgery (open reduction and internal fixation surgery for radius fractures or internal fixation taking out operation for radius fractures) completed within 2 h between January 2020 and December 2020 were enrolled. This study was approved by the Qingdao Municipal Hospital ethics committee and registered at the China Clinical Trials Register (No. ChiCTR1900028286, principal investigator: LX Sun, date of registration: 17 December 2019). Before the experiment, we obtained the informed consent of all enrolled patients and signed the consent form.

Exclusion criteria in this study were patients with lung diseases, nerve block failure, local anesthesia or neuropuncture contraindications, phrenic nerve abnormalities or diaphragm dysfunction, previous neck trauma or surgery history, rejected for participation in the study, pregnant or lactating women, obstructive sleep apnea syndrome, long-term use of pain medication, and mental, speech, or hearing impairment.

**Fig. 1:**
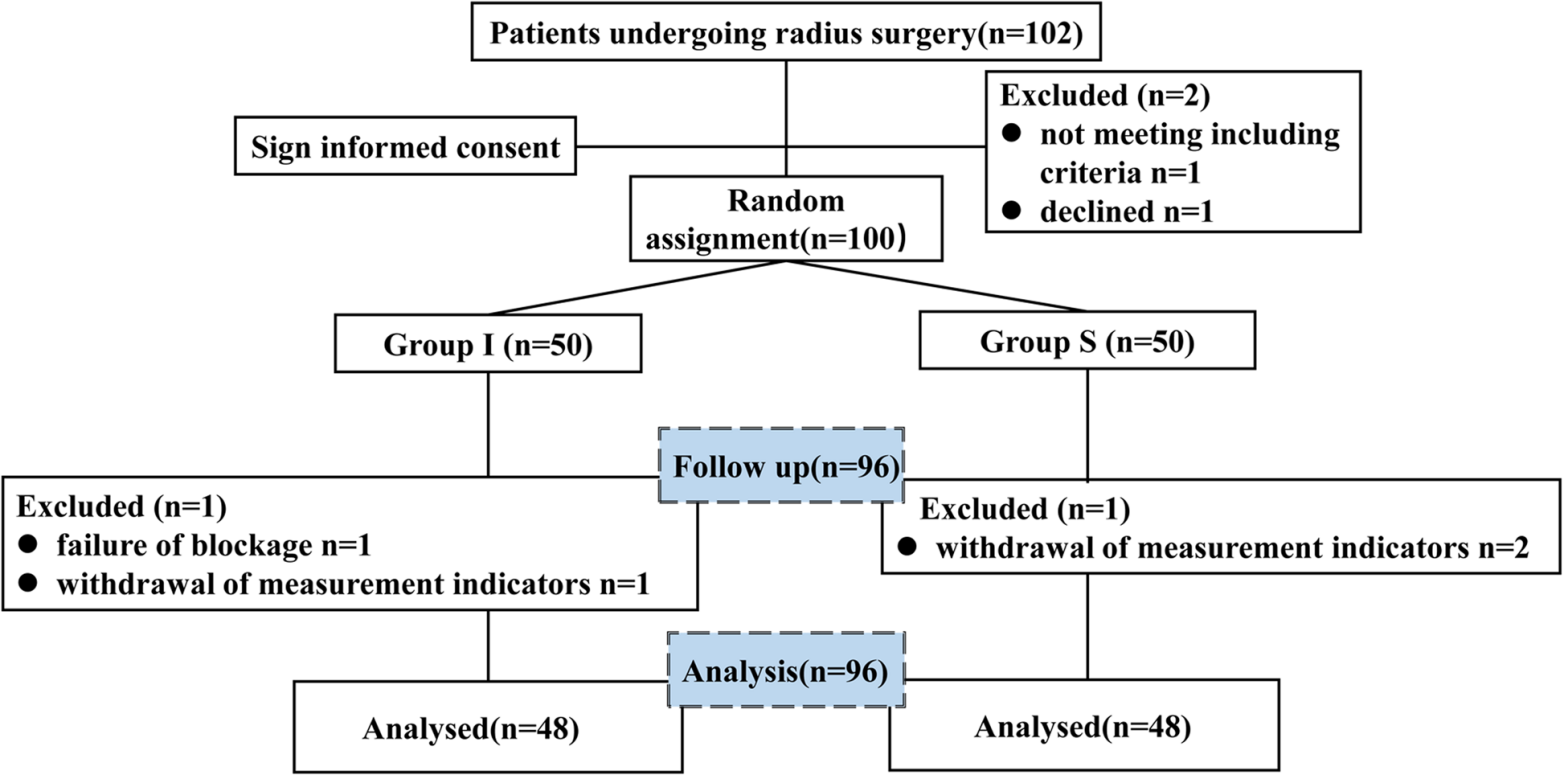
Consolidated Standards of Reporting Trials in this study

All enrolled patients were divided into two groups using a random number table method (computerized program) for group I and group S. An experienced anesthesiologist who was aware of the grouping performed the nerve blocks. Neither the patients nor the follow-up investigator knew the group assignment. This double-blind, randomized and controlled single-center study was implemented in accordance with the Helsinki declaration. Allocation of the patients was according to the trial flow diagram, which is presented in Fig. 1.

### The measurement of pre-block

All patients had routine fasting and no drinking before operation, no preoperative medication, and no oxygen was given. After entering the anesthesia preparation room, we open peripheral veins and connect BP, ECG, and SpO_2_ monitoring to patients.

Patients took sitting position and tested the lung function with a spirometer (HI-101, CHEST, Tokyo, Japan). The patient’s lung function indicators [Forced vital capacity (FVC), forced expiratory volume in 1 s (FEV_1_)] were recorded separately before anesthesia, and the average value was repeated three times.

The patients were placed in a supine position, using an ultrasound diagnostic apparatus and a 3–5 MHZ frequency convex array probe to measure the diaphragm mobility of the patients in the calm breathing position and the deep breathing position, respectively. Two-dimensional ultrasound mode was selected to place the probe between the midclavicular line and the anterior axillary line of the lower edge of the ipsilateral costal arch. On the basis of the liver or spleen being the acoustic window, an image of the diaphragm appeared as a hyperechoic band between the chest and abdomen. We adjusted the probe angle so that the sound beam was perpendicular to the movement direction of the diaphragm. The posterior 1/3 of the diaphragm was imaged. When the image was stable, we switched to M-mode ultrasound. The measurement standard was that the sampling line was perpendicular to the diaphragm muscle line and stable waveforms appeared [12]. We observed and recorded the amplitude of the 3 breathing cycles, and took the average as the result for the eupnea test. The movement of the diaphragm for deep breathing was the same as the above method [13].

The diaphragmatic movement reduction recorded in the two groups could be defined as not paralyzed, partially paralyzed, or completely paralyzed. Diaphragmatic movement reduction of less than 25% represented not paralyzed. Partially paralyzed was defined as a diaphragmatic movement reduction between 25% and 75%. Diaphragmatic movement reduction of more than 75% was seen as completely paralyzed [12, 14]. We calculated the incidence of diaphragmatic paralysis by summing the completely paralyzed and partially paralyzed.

### Ultrasound-guided ISB

Patients were placed in the supine position with the shoulders and arms naturally placed on both sides of the body and the head tilted to the opposite side from the surgical site. Then they were sedated with midazolam (0 to 20 mcg/kg) to achieve a Richmond Agitation-Sedation Scale score of − 2 to − 3. Skin disinfection was performed using betadine (Mingyaotang Pharmaceutical Technology Development Co., Ltd, Qingdao, China) and sterile drapes were applied according to the sterile principles guidelines. All blocks were completed by the same experienced anesthesiologist. A 5–12 MHZ high-frequency linear array probe was applied with ultrasonic coupling agent and then covered with a sterile membrane. After local infiltration anesthesia using 1% lidocaine 5 ml, a 22 G insulated puncture needle with 0.375% ropivacaine was applied. The ultrasound probe in group I was placed on the side of the neck to obtain a transverse view of the brachial plexus between the anterior scalene muscle and the middle scalene muscle. Cross-sectional imaging of the peripheral blood vessels and the position of the scalene muscle were used to determine the position of the brachial plexus trunk in the interscalene, which manifested as multiple circular or elliptical hypoechoic areas between the anterior scalene muscle and the middle scalene muscle, surrounded by a hyperechoic halo. The needle was inserted using an in-plane insertion technique, until the needle tip reached around upper trunk, middle trunk, and lower trunk [15]. After withdrawing the plunger of the syringe without blood, 15 ml of 0.375% ropivacaine was injected into the correct place.

### Ultrasound-guided SCB

The probe in group S was placed in the supraclavicular fossa to obtain a short-axis view of the subclavian artery. The brachial plexus appeared as a hyperechoic ring closely surrounding the subclavian artery and it formed a low-density grape-like structure. We advanced the puncture needle to the “corner pocket” (the angle between the first rib and the subclavian artery) by using an in-plane insertion technique [16]. The rest of the procedure was the same as in group I.

### Evaluation of block effects

The sensorimotor block was scored every 5 min within 30 min after the block by the same nursing staff who were unaware of the block method. The sensory block was evaluated in the C5 ~ T1 nerve trunk innervation area by a needle-punching method with the following score: 0 = normal pricking sensation, 1 = tactile presence and loss of pain, and 2 = tactile absence, and 1 and 2 points were recorded as a perfect block. The test points were as followed: inferior along the edge of the deltoid muscle (C5), the forearm to the radial side (C6), 2rd to 3rd interphalangeal of dorsal hand (C7), 4rd to 5rd interphalangeal of dorsal hand (C8), the inner forearm (T1). Motor block was evaluated by the operated arm lift and hand grip strength. The grading standards was followed: 0 = no motor block, 1 = feeling heavy, and 2 = unable to lift the limb or shake hands. 1 and 2 points were both recorded as a perfect block [12, 17]. If there was no perfect sensorimotor block within 30 min, the patient was excluded from the study.

### The measurement after block

Thirty minutes (T_30min_) after the injection, the degree of movement of the diaphragm during eupnea and deep breathing was measured and recorded by ultrasound. The indicators including FVC and FEV_1_ were measured using a spirometer. Besides, the analgesia score (VAS) during rest was assessed at thirty minutes (T_30min_) after blockage.

### General anesthesia

Subsequently, propofol (2 ~ 2.5 mg/kg) and cisatracurium besylate (0.15 mg/kg) were used for the induction of anesthesia before a laryngeal mask was applied. The mechanical ventilation settings were 6–8 ml/kg tidal volume and 10–12 times/min respiratory rate. Anesthesia was maintained with sevoflurane inhalation (≤ 1 MAC) and target-controlled infusion of propofol (30–50 µg/kg/min). The amount of anesthetic was appropriately adjusted to maintain an intraoperative end-tidal carbon dioxide partial pressure (P_ET_CO_2_) value of 35–45 mmHg and a bispectral index (BIS) value of 40–60. Propofol, ephedrine (5 mg), and atropine (0.3 mg) could be injected if the blood pressure increased > 20%, the blood pressure decreased > 20%, or the heart rate dropped < 50 beats/min. Each medication was recorded in detail. At the end of the operation, all patients received 5 mg tropisetron hydrochloride to prevent postoperative nausea and vomiting.

### The measurement of post-operative pulmonary function, adverse reactions, and VAS score

Thereafter, diaphragm movement was evaluated by ultrasound at eupnea and deep breathing at 4 (T_4_), 8 (T_8_), and 12 (T_12_) h after blockage. The incidence of diaphragmatic paralysis was calculated and recorded. Lung function (FEV_1_ and FVC) was measured at 4, 8, and 12 h. The blinded investigator recorded the occurrence of adverse reactions such as local anesthetic poisoning, Horner syndrome, vascular injury, pneumothorax, dyspnea, etc. within 12 h of blockage. After recovery in the post anesthesia care unit (PACU), the patients were transferred to the ward. The trained medical staff, unaware of the randomization, evaluated the recovery time from the sensory and motor block (the time from the end of the ropivacaine injection to recovery of pain sensation and movement). The VAS at rest was assessed at 4 (T_4_), 8 (T_8_), and 12 (T_12_) h after blockage. If the VAS score > 3 points, flurbiprofen axetil (50 mg) was administered for remedial analgesia within 12 h of blockage.

### Outcome measurement

The primary outcomes were diaphragm mobility (T_0_, T_30min_, T_4_, T_8,_ and T_12_), diaphragmatic paralysis rate (T_30min_, T_4_, T_8,_ and T_12_), and pulmonary function testing (FVC and FEV_1_) (T_0_, T_30min_, T_4_, T_8,_ and T_12_) in group I and group S. The secondary outcomes were: the VAS at rest (T_30min_, T_4_, T_8,_ and T_12_); the recovery time from the sensory and motor block; and adverse reactions within 12 h of blockage in each group.

### Statistical analysis

We used the PASS software (PASS 15. NCSS, USA) to calculate the total sample size with α = 0.05 (two-sided) and β = 0.1. Based on our pre-experimental results and previous literature [18], the sample size was calculated for each dependent primary outcomes and took the maximum sample size. Finally, the reference index for the sample size was the incidence of complete diaphragmatic paralysis, in which 78.75% of the interscalene block and 42.6% of the supraclavicular block [18]. *Fisher* exact test was performed for two proportions. The calculated number of each group was 42. To compensate for the loss to follow-up rate, we increased the sample size to 50. Two in the group I and two in the group S were excluded from study for failure of blockage and withdrawal of measurement indicators after surgery, which were described detailedly in Fig. 1. Finally, a total of 96 subjects were included in our study.

Statistical analysis adopted SPSS19.0 statistical software (IBM, Armonk, USA). The independent enumeration data were expressed as number of cases and percentage (%), and comparison between the two groups were analyzed with the Chi square or Chi square correction or Fisher’s exact test according to the condition of N and T. The independent approximately normal distribution measurement data (assessed with the Shapiro-Wilk test) was expressed as mean ± standard deviation and then analyzed with the independent-samples t test between the two groups. Extremely non-normally distributed data were expressed as median (25th to 75th percentile) and analyzed with the Mann-Whitney U test. The repeated normal distribution measurement data within the groups were analyzed by one-way repeated-measures ANOVA, with Bonferroni correction test was used to assess downstream time points (T_30min_, T_4_, T_8_, and T_12)_ versus initial time point (T_0_). If the result did not meet the sphericity test, then we refer to the test of Greenhouse-Geisser. For the sake of outliers, a test of normality was performed. Considering the robustness of one-way repeated-measures ANOVA, individual outliers were included in the statistical analysis, when detected. *p* < 0.05 was considered to be a significant difference.

## Results

Patient’s general information are shown in Table 1. Among the 96 patients enrolled, there was no statistical difference between baseline materials of the 2 groups of patients. All patients had complete sensory and motor disturbances after 30 min of block, after the block was finished, we performed general anesthesia using laryngeal mask for all patients in order to eliminate the patient’s tension. Before operation, there were no significant differences in diaphragm mobility and lung function between the patients in group S and group I.

**Table 1:** Baseline materials

	group I (*n*=48)	group S (*n*=48)	*p* value
Age (yr,$\overline x$ ±s )	46±6	47±10	0.891
Weight (kg,$\overline x$ ±s)	67±7	70±8	0.575
Height (cm,$\overline x$ ±s)	170±8	171±7	0.292
BMI (kg/m^2^,$\overline x$ ±s)	23.5 (22.5-25)	23.4 (21.4-24.7)	0.101
Sex (male/female)	27/21	25/23	0.682
Side of block (right/left)	23/25	28/20	0.306
Duration of surgery (min,$\overline x$ ±s)	85±16	82±16	
Radius fracture surgery			
ORIF/IFTO	27/21	24/24	0.539
Diaphragm mobility			
Eupnea breathing of T_0_ (cm)	2.03±0.24	1.97±0.24	0.184
Deep breathing of T_0_ (cm)	5.71±0.43	5.67±0.50	0.617
FEV_1_ of T_0_ (L)	2.90±0.27	3.00±0.33	0.095
FVC of T_0_( L)	3.47±0.33	3.60±0.41	0.093

Values are expressed as mean ± standard deviation, or absolute numbers.

*BMI* Body mass index, *FVC* Forced vital capacity, *FEV*_*1*_ Forced expiratory volume in 1 s, *ORIF *Open reduction and internal fixation surgery for radius fractures, *IFTO* Internal fixation taking out operation for radius fractures.

**Fig. 2:**
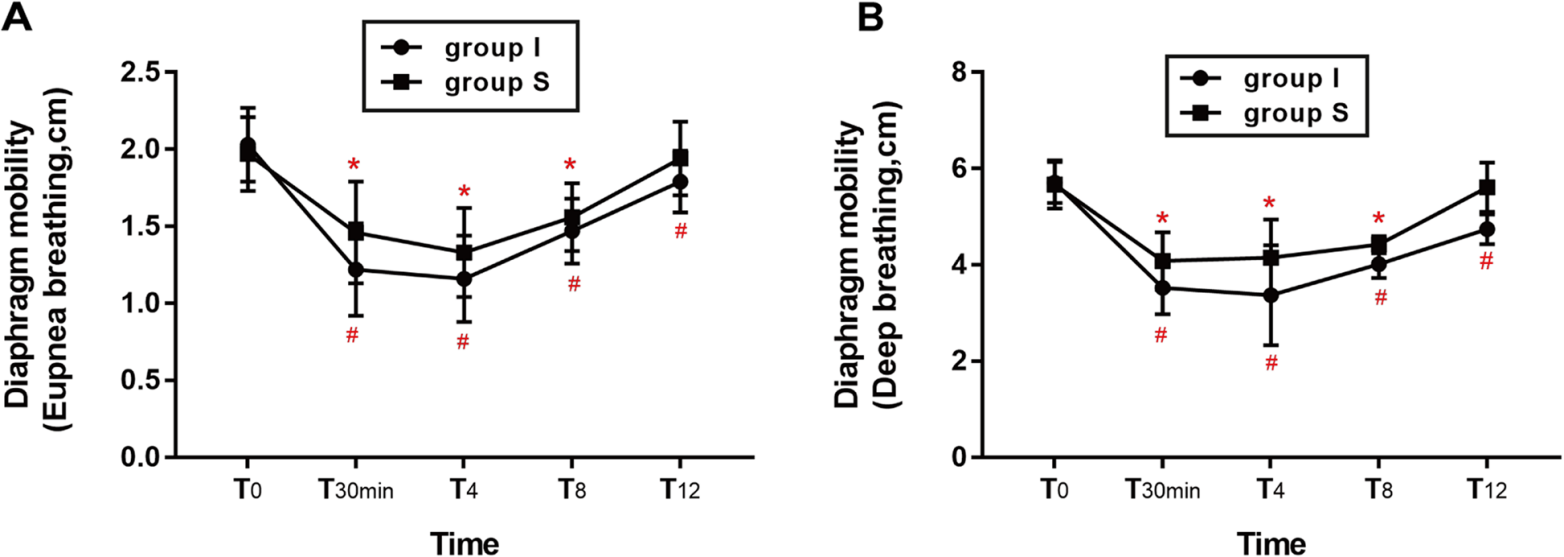
Diaphragm mobility. Data are expressed as mean ± standard deviation. One-way repeated measures ANOVA with Bonferroni post hoc was employed in the within group timepoints comparison. In group S, *p < 0.01 when T_12_ compared with T_0_; In group I, #*p* < 0.01 when T_12_ compared with T_0_

One-way repeated measures ANOVA was employed in diaphragm mobility of eupnea breathing and deep breathing. As shown in Fig. 2, the within group timepoints comparison showed no statistically differences in group S between T_0_ and T_12_ (1.97 ± 0.24 vs. 1.94 ± 0.24, *p* = 0.175; 5.67 ± 0.50 vs. 5.61 ± 0.51, *p* = 0.105). However, this result did not arise in the group I (2.03 ± 0.24 vs. 1.79 ± 0.20, *p* < 0.001; 5.71 ± 0.43 vs. 4.74 ± 0.31, *p* < 0.001).

The incidence of diaphragmatic paralysis in eupnea breathing and deep breathing was also lower in group S compared with group I comprising the three timepoints of measurement [T_30min_ (65% vs. 38%, *p* = 0.008), T_4_ (79% vs. 46%, *p* = 0.001), T_8_ (54% vs. 17%, *p* < 0.001)]. Difference not statistically significant within T_12_ between the two groups [T_12_ (8% vs. 0%, *p* = 0.117)]. The incidence of diaphragmatic paralysis in deep breathing of group S indeed decreased significantly during T_30min_, T_4,_ T_8_, and T_12_ [T_30min_ (77% vs. 48%, *p* = 0.003), T_4_ (67% vs. 33%, *p* = 0.001), T_8_ (52% vs. 29%, *p* = 0.022),T_12_ (21% vs. 0%, *p* = 0.001)]than group I (Table 2).

**Table 2:** Incidence of phrenic nerve block

			group I (*n*=48)	group S (*n*=48)	*p* value
Incidence of phrenic nerve block (%)	Eupnea breathing	T_30min_	31 (65)	18 (38)	0.008
		T_4_	38 (79)	22 (46)	0.001
		T_8_	26 (54)	8 (17)	<0.001
		T_12_	4 (8)	0	0.117
	Deep breathing	T_30min_	37 (77)	23 (48)	0.003
		T_4_	32 (67)	16 (33)	0.001
		T_8_	25 (52)	14 (29)	0.022
		T_12_	10 (21)	0	0.001

Values are expressed as number (percent). The incidence of diaphragmatic paralysis in eupnea breathing and deep breathing were analyzed with the Chi square or Chi square correction or Fisher’s exact test according to the condition of N and T.

**Fig. 3:**
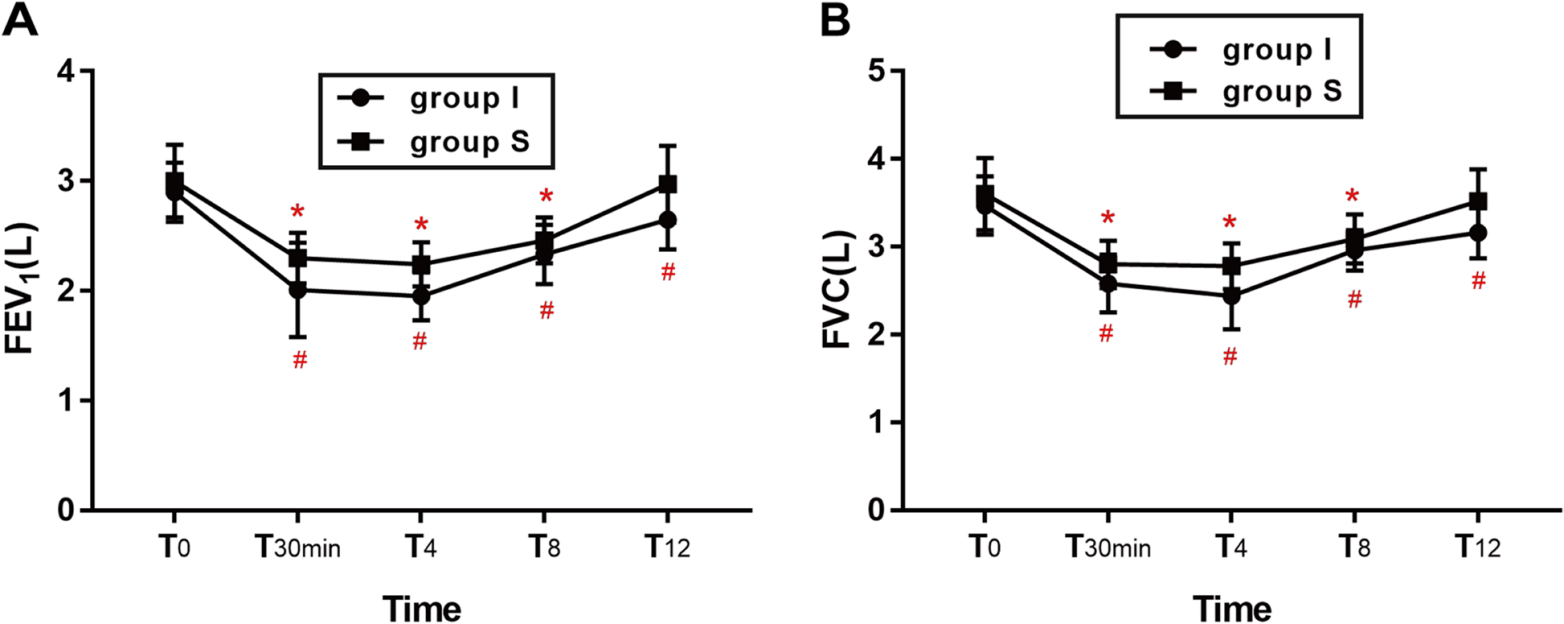
Lung function (FEV_1_ and FVC). Data are expressed as mean ± standard deviation. One-way repeated measures ANOVA with Bonferroni post hoc was employed in the within group timepoints comparison. In group S, *p < 0.01 when T_12_ compared with T_0_; In group I, #*p* < 0.01 when T_12_ compared with T_0_; FVC, forced vital capacity; FEV_1_, 1 s expiratory volume

One-way repeated measures ANOVA was employed in FEV_1_ and FVC between the two groups. Intergroup comparison showed no statistically differences in group S between T_0_ and T_12_ (3.00 ± 0.33 vs. 2.97 ± 0.35, *p* = 0.058; 3.60 ± 0.41 vs. 3.52 ± 0.36, *p* = 0.376). The FEV_1_ and FVC of group I confirmed an absence of a statistically significant difference between T_0_ and T_12_ (2.90 ± 0.27 vs. 2.65 ± 0.27, *p* = 0.000; 3.47 ± 0.33 vs. 3.16 ± 0.29, *p* = 0.000) (Fig. 3).

There is no statistical significant difference with the VAS score at each time point between the two groups, as expected (Table 3). The recovery time of sensory and motor block did not appear different between group I and group S (Table 4). Likewise, adverse reactions did not differ between the 2 groups (Table 5).

**Table 3:** VAS at rest

	group I (*n* = 48)	group S (*n* = 48)	*p* value
analgesia score (VAS at rest)			
T_30min_	0(0–0)	0(0–0)	0.684
T_4_	0(0–0)	0(0–0)	0.557
T_8_	0(0–1)	0(0–0)	0.814
T_12_	0(0–1)	0(0–0)	0.961

Values are expressed as median (interquartile range), *VAS* Visual analogue scale

**Table 4:** The recovery time of sensory and motor block

	group I (*n* = 48)	group S (*n* = 48)	*p* value
sensory recovery time (h)	11.6(10.9-12.8)	11.5(10.5–12.2)	0.224
motor recovery time (h)	9.8(9.0–11.0)	9.5(8.7–10.0)	0.206

Values are expressed as median (interquartile range)

**Table 5:** The incidences of adverse events within 12 h of blockage in two groups

	group I (*n* = 48)	group S (*n* = 48)	*p* value
remedial analgesia (N(%))	2 (4)	3(6)	> 0.999
Adverse reactions			
Local anesthetic poisoning	0	0	-
Horner syndrome	5(10)	4(8)	> 0.999
Vascular injury	0	0	-
Pneumothorax	0	0	-
Dyspnea	0	0	-
Vomit	2(4)	2(4)	> 0.999
Nausea	2(4)	1(2)	> 0.999

Values are expressed as number (percentage)

## Discussion

Brachial plexus block, a kind of local anesthetic, is effective in shoulder and upper limb surgery with or without general anesthesia [19]. It could not only reduce postoperative opioid consumption, but also effectively promote rapid postoperative recovery and reduce the hospital length of stay [20]. The SCB and ISB are the most frequently used nerve blocks, although diaphragm paralysis is a known complication of both blocks [18]. Since the interscalene plexus is very close to the phrenic nerve at a high level of the pouch, the risk of diaphragm paralysis is higher [21]. Also correlation between the occurrence of diaphragm paralysis and supraclavicular plexus block has been observed [22]. This is mainly related to the contralesional diffusion of local anesthetics and applying pressure on the proximal nerve trunk does not contribute to reducing the paralysis rate. Respiratory function during the early postoperative course, comparing supraclavicular plexus block and interscalene plexus block has rarely been studied or investigated.

The brachial plexus is close to the abdominal branch of the C5 nerve at the cricoid cartilage, with an average distance of 1.8 mm. On average, the distance between the brachial plexus and phrenic nerve increases by 3 mm for every 1 cm of the neck to the caudal end. Thus, it is easy to infer that the phrenic nerve is highly vulnerable to also be blocked during an ISB. Diaphragm mobility is first affected, then the blockage involves the lung function. Our preliminary study showed that the diaphragm mobility of group I was lower than that of group S at T_30min_, T_4_, T_8_, and T_12_, which may be related to the position of the phrenic nerve. At the same time, the diaphragm mobility of group S was not statistically different between the level before the block T_0_ and T_12_, but the difference was significant in group I. In terms of the recovery speed of diaphragm mobility, the results implied that the changes of diaphragm mobility in group S returned to a near-normal level after a 12-hour recovery time; this phenomenon, however, was absent in group I, which provided a reference for blinding postoperative observation period. This was probably related to the recovery time of the sensory block, but, as a whole, the recovery of diaphragm mobility was delayed. Many inherent causes may have contributed: uncontrollable mechanical damage to the phrenic nerve, the toxicity of anesthetics, the vasoconstrictor effect of ropivacaine, and so on. We used ultrasound technology, the most common evaluation tool for monitoring diaphragm function [23], to quantitatively measure the movement of the diaphragm before and after brachial plexus block and analyzed the incidence of phrenic nerve block. Due to the obstruction of the spleen and gastrointestinal cavity organs on the left side of the diaphragm, the diaphragm had an unclear outline, making it difficult to measure the movement of the left side of the diaphragm [24]. It is easier to observe the movement of the diaphragm through the right side. In order to approximate the actual clinical situation, patients with right brachial plexus block were not specifically selected.

The main observation indicators of our experiment indicated that the incidence of the phrenic nerve block of group I was 67%, and the rate of diaphragmatic palsy of group S was 41%. Incidence of HDP after ISB from our study was lower than previous studies [22, 25]. This may be due to different kind, concentrations, and doses of local anesthetics. Although our study had a small study sample, this is consistent with other studies. This may not be sufficient to develop respiratory distress with accessory respiratory muscles assisting [26]. Besides, a previous study has suggested that the movement of the contralateral diaphragm can be compensated for synchronously with the change in the movement of the blocked diaphragm according to contralateral diaphragm assessment [27]. However, due to the delayed diaphragmatic palsy, the block may have serious consequences if it was implemented in patients with respiratory diseases or obesity [28, 29]. When not supported by adequate supervision, patients with respiratory depression may be ignored.

Decreased diaphragm activity affects the patient’s breathing. The invasive operation of blood gas analysis of patients during a short operation time is not in line with the principle of benefiting them. Therefore, we evaluated the patient’s respiratory function by non-invasive lung function measurements. General anesthesia was applied to these patients after the nerve block, which might increase patient comfort. Since the pulse oximetry were affected by general anesthesia, we do not include it in the detection index. Other studies have shown that as long as the patients were in good physical condition, unilateral phrenic nerve block was less likely to cause a severe drop in pulse oximetry [13, 22].

Pulmonary complications mostly occur in the postoperative period. Our results showed that pulmonary function testing (FVC and FEV_1_) of both groups was impaired to different extents after the blocks. Diaphragmatic paralysis could not increase the transverse and anteroposterior diameter of the thoracic cavity, so that the volume of the lungs could not be increased correspondingly [30]. In the one-way repeated-measures ANOVA analysis, FVC and FEV_1_ of group S was recovered after 12 h of blockade in our study, while in group I they were not, which seemed to go hand in hand with diaphragm mobility. The change of lung function may be related to the degree of diaphragm paralysis [31, 32]. Although possibly related to regional anesthesia, ventilation under general anesthesia could also be associated with exposure to lung injury. However, this should have little effect on the results, since it should be equally distributed between group S and group I.

The differences in analgesic effect just failed to reach statistical significance at T_30min_, T_4_, T_8_, and T_12_. The recovery of motor and sensorimotor functions was not different between the two groups. We found no significant difference in the adverse events between the two groups, even though Horner syndrome, nausea, and vomiting had a trend toward a difference between the two groups. We hypothesized that the incidence of nausea and vomiting may be affected by the anesthesia induction was performed by endotracheal intubation which can bias these results.

Our study has several limitations. Considering compliance to the treatment of the patients, our measurements pertained only to the 12 h postoperative period, and thus the recovery time of respiratory function in group I needs to be explored in larger, longer-term studies. Besides, local anesthetic volume and needle type were also not taken into account.

## Conclusions

Overall, our study provided plausible findings that the recovery duration of respiratory function (diaphragm mobility, diaphragmatic paralysis rate, and pulmonary function testing) after SCB (12 h) is shorter than those after ISB (> 12 h), which provides a reference for postoperative support and monitoring and increases the safety of the surgical procedure.

## Data Availability

The datasets are available from the corresponding author on reasonable request.
